# 3,5-Bis(3-butylimidazolium-1-ylmethyl)toluene bis(hexafluorophosphate)

**DOI:** 10.1107/S1600536810040055

**Published:** 2010-10-13

**Authors:** Rosenani A. Haque, Abbas Washeel, Siang Guan Teoh, Ching Kheng Quah, Hoong-Kun Fun

**Affiliations:** a School of Chemical Sciences, Universiti Sains Malaysia, 11800 USM, Penang, Malaysia; b X-ray Crystallography Unit, School of Physics, Universiti Sains Malaysia, 11800 USM, Penang, Malaysia

## Abstract

In the title compound [systematic name: 3,3′-Dibutyl-1,1′-(5-methyl-*m*-phenyl­enedimethyl­ene)diimidazol-1-ium bis­(hexa­fluoridophosphate)], C_23_H_34_N_4_^2+^·2PF_6_^−^, the imidazole rings are inclined at angles of 68.06 (7) and 75.05 (8)° with respect to the central benzene ring. In the crystal, mol­ecules are linked into one-dimensional columns along [010] *via* weak inter­molecular C—H⋯F hydrogen bonds. The crystal structure is further consolidated by weak C—H⋯π(arene) inter­actions. One of the *n*-butyl groups is disordered over two sites with refined occupancies of 0.694 (5) and 0.306 (5). In addition, four of the F atoms of one of the PF_6_^−^ cations are disordered over two sites with occupancies of 0.64 (3) and 0.36 (3).

## Related literature

For general background to imidazoline-2-ylidenes, see: Arduengo *et al.* (1991 ▶). For the organometallic and coordin­ation chemistry of *N*-heterocyclic carbene ligands, see: Chen *et al.* (2002 ▶); Zhou *et al.* (2008 ▶); Hahn & Jahnke (2008 ▶); Danopoulos *et al.* (2007 ▶); Bourissou *et al.* (2000 ▶); McGuinness & Cavell (2000 ▶); Garrison *et al.* (2001 ▶). For catalytic studies related to organic synthesis, see: Cavell & McGuinness (2004 ▶); Liu *et al.* (2007 ▶). For the stability of the temperature controller used in the data collection, see: Cosier & Glazer (1986 ▶). For standard bond-length data, see: Allen *et al.* (1987 ▶).
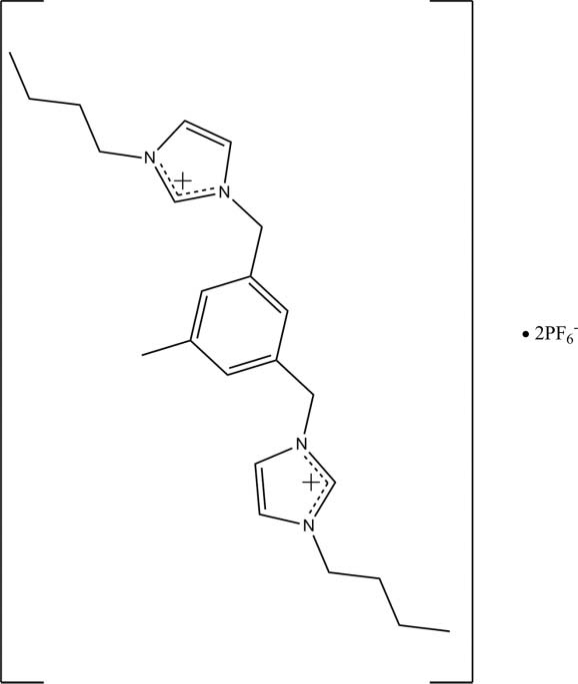


## Experimental

### 

#### Crystal data

C_23_H_34_N_4_^2+^·2PF_6_^−^
*M* *_r_* = 656.48
Monoclinic, 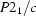

*a* = 9.6207 (1) Å
*b* = 11.1801 (1) Å
*c* = 27.9277 (3) Å
β = 102.416 (1)°
*V* = 2933.66 (5) Å^3^
*Z* = 4
Mo *K*α radiation
μ = 0.25 mm^−1^
*T* = 100 K
0.49 × 0.20 × 0.14 mm

#### Data collection

Bruker SMART APEXII CCD area-detector diffractometer
Absorption correction: multi-scan (*SADABS*; Bruker, 2009 ▶) *T*_min_ = 0.890, *T*_max_ = 0.967
45569 measured reflections
10399 independent reflections
7294 reflections with *I* > 2σ(*I*)
*R*_int_ = 0.040

#### Refinement

*R*[*F* ^2^ > 2σ(*F* ^2^)] = 0.046
*wR*(*F* ^2^) = 0.122
*S* = 1.03
10399 reflections
448 parameters
H-atom parameters constrained
Δρ_max_ = 0.39 e Å^−3^
Δρ_min_ = −0.26 e Å^−3^

### 

Data collection: *APEX2* (Bruker, 2009 ▶); cell refinement: *SAINT* (Bruker, 2009 ▶); data reduction: *SAINT*; program(s) used to solve structure: *SHELXTL* (Sheldrick, 2008 ▶); program(s) used to refine structure: *SHELXTL*; molecular graphics: *SHELXTL*; software used to prepare material for publication: *SHELXTL* and *PLATON* (Spek, 2009 ▶).

## Supplementary Material

Crystal structure: contains datablocks global, I. DOI: 10.1107/S1600536810040055/lh5143sup1.cif

Structure factors: contains datablocks I. DOI: 10.1107/S1600536810040055/lh5143Isup2.hkl

Additional supplementary materials:  crystallographic information; 3D view; checkCIF report

## Figures and Tables

**Table 1:** Hydrogen-bond geometry (Å, °) *Cg*1 is the centroid of the C1–C6 phenyl ring.

*D*—H⋯*A*	*D*—H	H⋯*A*	*D*⋯*A*	*D*—H⋯*A*
C8—H8*A*⋯F4	0.93	2.42	3.0154 (16)	121
C8—H8*A*⋯F5	0.93	2.39	3.2700 (16)	157
C16—H16*A*⋯F3	0.93	2.31	3.1941 (16)	160
C16—H16*A*⋯F4	0.93	2.45	3.1677 (16)	134
C21*A*—H21*B*⋯F4	0.97	2.49	3.196 (2)	130
C3—H3*A*⋯F12^i^	0.93	2.44	3.3147 (16)	157
C5—H5*A*⋯F9*A*^ii^	0.93	2.55	3.420 (8)	156
C7—H7*A*⋯F10*A*^ii^	0.97	2.51	3.427 (6)	158
C9—H9*A*⋯F12^iii^	0.93	2.48	3.2805 (17)	144
C12—H12*A*⋯F3^iv^	0.97	2.53	3.3358 (18)	140
C18—H18*A*⋯F8*A*^v^	0.93	2.38	3.148 (11)	140
C22*A*—H22*C*⋯*Cg*1^vi^	0.96	2.76	3.535 (3)	138
C23—H23*B*⋯*Cg*1^vii^	0.96	2.59	3.487 (2)	155
C22*B*—H22*E*⋯*Cg*1^vi^	0.96	2.86	3.637 (8)	139

Symmetry codes: (i) 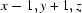; (ii) 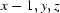; (iii) 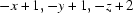; (iv) 
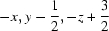
; (v) 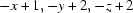; (vi) 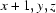; (vii) 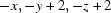.

**Fig. 1.**
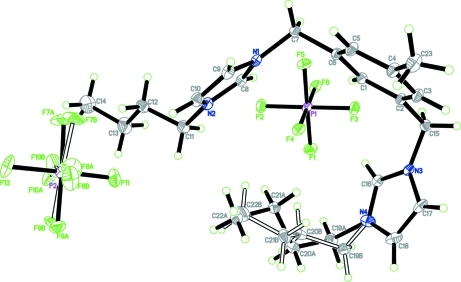
The molecular structure of the title compound showing 30% probability displacement ellipsoids for non-H atoms and the atom-numbering scheme. Both disorder components are shown.

**Fig. 2.**
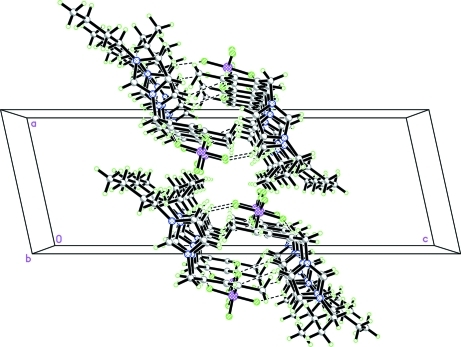
Part of the crystal structure of the title compound, viewed along the b axis. Only the major disorder component is shown. Intermolecular hydrogen bonds are shown in dashed lines.

## supplementary crystallographic information

### Comment

Since the discovery of stable imidazoline-2-ylidenes, which were isolated and structurally characterized by Arduengo *et al.* (1991), the organometallic and coordination chemistry of *N*-heterocyclic carbene (NHC) ligands have been receiving great attention in recent years and much interest has been generated in the chemistry of the metal complexes of these ligands (Chen *et al.*, 2002; Zhou *et al.*, 2008). NHC carbene, a strong σ-donor and a weak π-acceptor, strongly interacts with different transitions metals in various oxidation states (Hahn & Jahnke, 2008; Danopoulos *et al.*, 2007). Heterocyclic carbenes derived from imidazolium ions form complexes with many transition metals; heterocyclic carbene complexes of Pd, Ni, Pt, Rh, Ru, Ag and Au have been reported (Bourissou *et al.*, 2000; McGuinness & Cavell, 2000; Garrison *et al.*, 2001). Extensive catalytic studies on the application to organic synthesis have also been reported (Cavell & McGuinness, 2004; Liu *et al.*, 2007).

The title molecule (Fig. 1) consists of a 3,5-bis(3-butylimidazolium-1- ylmethyl)toluene cation and two hexafluorophosphate anions. Bond lengths (Allen *et al.*, 1987) and angles are within normal ranges.The phenyl ring (C1-C6) is inclined at angles of 68.06 (7) and 75.05 (8) ° with respect to the N1/N2/C8/C9/C10 and N3/N4/C16/C17/C18 imidazole rings. The butyl group (C19-C22) of the cation and four fluorine atoms (F7-F10) of the hexafluorophosphate anion are disordered over two positions with refined site-occupancies of 0.694 (5) : 0.306 (5) and 0.64 (3) : 0.36 (3), respectively.

In the crystal structure, (Fig. 2), the molecules are linked into one-dimensional columns along [010] via intermolecular C–H···F hydrogen bonds (Table 1). The crystal structure is further consolidated by C–H···Cg1 (Table 1) interactions, Cg1 is the centroid of C1-C6 phenyl ring.

### Experimental

*N*-butylimidazole (0.9 g, 7.2 mmol) was added to a stirred solution of 3,5-bis(bromomethyl)toluene (1.0 g, 3.6 mmol) in 20 ml of 1,4-dioxane. The mixture was refluxed at 373 K for 24 h. The sticky product was isolated by decantation and washed with fresh 1,4-dioxane (2 x 5 ml) and diethyl ether (2 x 3 ml). The resulting bromide salt was converted directly to its hexafluorophosphate salt by metathesis reaction using KPF_6_ (1.3 g, 7.2 mmol) in 20 ml of methanol. The colourless precipitate formed was collected and washed with distilled water (2 x 5 ml), and then recrystallized from acetonitrile to give colourless crystals. Yield: 1.9 g (79%), *m. p.*: 369-371 K. Crystals suitable for X-ray diffraction studies were obtained by slow evaporation of the salt solution in acetonitrile at 281 K.

### Refinement

All H atoms were positioned geometrically and refined using a riding model with C–H = 0.93-0.97 Å and *U*_iso_(H) = 1.2 or 1.5 *U*_eq_(C). A rotating-group model was applied for the methyl groups. Butyl group (C19-C22) of cation and four fluorine atoms (F7-F10) of the hexafluorophosphate anion are disordered over two positions with refined site-occupancies of 0.694 (5) : 0.306 (5) and 0.64 (3) : 0.36 (3), respectively.

### Crystal data

**Table:**

C_23_H_34_N_4_^2+^·2PF_6_^−^	*F*(000) = 1352
*M**_r_* = 656.48	*D*_x_ = 1.486 Mg m^−^^3^
Monoclinic, *P*2_1_/*c*	Mo *K*α radiation, λ = 0.71073 Å
Hall symbol: -P 2ybc	Cell parameters from 9893 reflections
*a* = 9.6207 (1) Å	θ = 2.2–32.2°
*b* = 11.1801 (1) Å	µ = 0.25 mm^−^^1^
*c* = 27.9277 (3) Å	*T* = 100 K
β = 102.416 (1)°	Block, colourless
*V* = 2933.66 (5) Å^3^	0.49 × 0.20 × 0.14 mm
*Z* = 4	

### Data collection

**Table:**

Bruker SMART APEXII CCD area-detector diffractometer	10399 independent reflections
Radiation source: fine-focus sealed tube	7294 reflections with *I* > 2σ(*I*)
graphite	*R*_int_ = 0.040
φ and ω scans	θ_max_ = 32.3°, θ_min_ = 2.0°
Absorption correction: multi-scan (*SADABS*; Bruker, 2009)	*h* = −14→14
*T*_min_ = 0.890, *T*_max_ = 0.967	*k* = −15→16
45569 measured reflections	*l* = −41→41

### Refinement

**Table:**

Refinement on *F*^2^	Primary atom site location: structure-invariant direct methods
Least-squares matrix: full	Secondary atom site location: difference Fourier map
*R*[*F*^2^ > 2σ(*F*^2^)] = 0.046	Hydrogen site location: inferred from neighbouring sites
*wR*(*F*^2^) = 0.122	H-atom parameters constrained
*S* = 1.03	*w* = 1/[σ^2^(*F*_o_^2^) + (0.0569*P*)^2^ + 0.5709*P*] where *P* = (*F*_o_^2^ + 2*F*_c_^2^)/3
10399 reflections	(Δ/σ)_max_ = 0.001
448 parameters	Δρ_max_ = 0.39 e Å^−^^3^
0 restraints	Δρ_min_ = −0.26 e Å^−^^3^

### Special details

**Table:**

Experimental. The crystal was placed in the cold stream of an Oxford Cyrosystems Cobra open-flow nitrogen cryostat (Cosier & Glazer, 1986) operating at 100.0 (1) K.
Geometry. All esds (except the esd in the dihedral angle between two l.s. planes) are estimated using the full covariance matrix. The cell esds are taken into account individually in the estimation of esds in distances, angles and torsion angles; correlations between esds in cell parameters are only used when they are defined by crystal symmetry. An approximate (isotropic) treatment of cell esds is used for estimating esds involving l.s. planes.
Refinement. Refinement of F^2^ against ALL reflections. The weighted R-factor wR and goodness of fit S are based on F^2^, conventional R-factors R are based on F, with F set to zero for negative F^2^. The threshold expression of F^2^ > 2sigma(F^2^) is used only for calculating R-factors(gt) etc. and is not relevant to the choice of reflections for refinement. R-factors based on F^2^ are statistically about twice as large as those based on F, and R- factors based on ALL data will be even larger.

### Fractional atomic coordinates and isotropic or equivalent isotropic displacement parameters (Å^2^)

**Table:**

	*x*	*y*	*z*	*U*_iso_*/*U*_eq_	Occ. (<1)
N1	0.04726 (12)	0.72856 (9)	0.87680 (4)	0.0197 (2)	
N2	0.25427 (13)	0.70746 (10)	0.85917 (4)	0.0242 (2)	
N3	0.01110 (12)	1.25423 (9)	0.87615 (4)	0.0192 (2)	
N4	0.22616 (13)	1.27303 (12)	0.86629 (5)	0.0289 (3)	
C1	−0.12271 (14)	0.98508 (11)	0.87153 (5)	0.0201 (3)	
H1A	−0.1079	0.9856	0.8397	0.024*	
C2	−0.14332 (14)	1.09268 (11)	0.89438 (5)	0.0203 (2)	
C3	−0.16796 (15)	1.09046 (12)	0.94182 (5)	0.0241 (3)	
H3A	−0.1824	1.1620	0.9570	0.029*	
C4	−0.17140 (17)	0.98323 (12)	0.96688 (6)	0.0262 (3)	
C5	−0.14902 (16)	0.87688 (12)	0.94368 (5)	0.0257 (3)	
H5A	−0.1506	0.8047	0.9601	0.031*	
C6	−0.12428 (14)	0.87685 (11)	0.89618 (5)	0.0209 (3)	
C7	−0.10445 (14)	0.75962 (12)	0.87112 (5)	0.0230 (3)	
H7A	−0.1524	0.6963	0.8850	0.028*	
H7B	−0.1480	0.7657	0.8365	0.028*	
C8	0.12152 (15)	0.74404 (12)	0.84230 (5)	0.0219 (3)	
H8A	0.0861	0.7755	0.8112	0.026*	
C9	0.13609 (16)	0.67800 (12)	0.91727 (5)	0.0258 (3)	
H9A	0.1115	0.6571	0.9466	0.031*	
C10	0.26525 (16)	0.66465 (13)	0.90628 (5)	0.0272 (3)	
H10A	0.3462	0.6327	0.9265	0.033*	
C11	0.36527 (17)	0.70733 (14)	0.83004 (6)	0.0322 (3)	
H11A	0.4518	0.7418	0.8495	0.039*	
H11B	0.3345	0.7573	0.8013	0.039*	
C12	0.39714 (16)	0.58281 (14)	0.81384 (6)	0.0296 (3)	
H12A	0.3086	0.5432	0.7991	0.035*	
H12B	0.4430	0.5366	0.8423	0.035*	
C13	0.4934 (2)	0.58630 (18)	0.77702 (8)	0.0466 (5)	
H13A	0.4506	0.6373	0.7497	0.056*	
H13B	0.5843	0.6210	0.7926	0.056*	
C14	0.5180 (2)	0.4634 (2)	0.75778 (8)	0.0551 (5)	
H14A	0.5779	0.4700	0.7345	0.083*	
H14B	0.4283	0.4289	0.7421	0.083*	
H14C	0.5633	0.4133	0.7845	0.083*	
C15	−0.13694 (14)	1.21063 (12)	0.86830 (5)	0.0223 (3)	
H15A	−0.1752	1.2004	0.8335	0.027*	
H15B	−0.1949	1.2693	0.8805	0.027*	
C16	0.09876 (15)	1.22892 (12)	0.84694 (5)	0.0222 (3)	
H16A	0.0747	1.1869	0.8176	0.027*	
C17	0.08522 (17)	1.31852 (12)	0.91586 (5)	0.0270 (3)	
H17A	0.0493	1.3482	0.9419	0.032*	
C18	0.21968 (18)	1.32975 (14)	0.90954 (6)	0.0323 (3)	
H18A	0.2942	1.3686	0.9305	0.039*	
C19A	0.3539 (4)	1.2443 (3)	0.84464 (16)	0.0311 (7)	0.694 (5)
H19A	0.4058	1.3175	0.8418	0.047*	0.694 (5)
H19B	0.3214	1.2117	0.8120	0.047*	0.694 (5)
C20A	0.4534 (3)	1.1550 (2)	0.87594 (12)	0.0333 (7)	0.694 (5)
H20A	0.4875	1.1894	0.9082	0.040*	0.694 (5)
H20B	0.5352	1.1428	0.8614	0.040*	0.694 (5)
C21A	0.3866 (2)	1.0333 (2)	0.88191 (8)	0.0271 (6)	0.694 (5)
H21A	0.3007	1.0451	0.8942	0.041*	0.694 (5)
H21B	0.3600	0.9950	0.8501	0.041*	0.694 (5)
C22A	0.4863 (3)	0.9528 (3)	0.91641 (12)	0.0333 (6)	0.694 (5)
H22A	0.4374	0.8806	0.9215	0.040*	0.694 (5)
H22B	0.5185	0.9928	0.9472	0.040*	0.694 (5)
H22C	0.5666	0.9335	0.9025	0.040*	0.694 (5)
C19B	0.3518 (10)	1.2886 (6)	0.8487 (4)	0.0292 (15)	0.306 (5)
H19C	0.4196	1.3384	0.8707	0.044*	0.306 (5)
H19D	0.3316	1.3243	0.8163	0.044*	0.306 (5)
C20B	0.4073 (6)	1.1615 (5)	0.8473 (2)	0.0280 (13)	0.306 (5)
H20C	0.4935	1.1632	0.8346	0.034*	0.306 (5)
H20D	0.3372	1.1145	0.8249	0.034*	0.306 (5)
C21B	0.4395 (6)	1.1003 (5)	0.8976 (2)	0.0306 (14)	0.306 (5)
H21C	0.5222	1.1373	0.9183	0.046*	0.306 (5)
H21D	0.3594	1.1108	0.9131	0.046*	0.306 (5)
C22B	0.4668 (8)	0.9706 (7)	0.8925 (4)	0.049 (2)	0.306 (5)
H22D	0.4743	0.9320	0.9237	0.058*	0.306 (5)
H22E	0.5541	0.9602	0.8816	0.058*	0.306 (5)
H22F	0.3898	0.9359	0.8691	0.058*	0.306 (5)
C23	−0.2051 (2)	0.98208 (14)	1.01718 (6)	0.0348 (4)	
H23A	−0.2508	1.0557	1.0225	0.052*	
H23B	−0.1185	0.9735	1.0415	0.052*	
H23C	−0.2673	0.9162	1.0196	0.052*	
P1	0.03700 (4)	0.98649 (3)	0.736481 (13)	0.02052 (8)	
F1	0.14427 (10)	1.08561 (8)	0.72469 (3)	0.0333 (2)	
F2	0.13549 (10)	0.88117 (8)	0.72386 (3)	0.0322 (2)	
F3	−0.06262 (9)	1.09104 (7)	0.75081 (3)	0.02749 (18)	
F4	0.12345 (10)	0.98346 (7)	0.79273 (3)	0.0288 (2)	
F5	−0.07193 (9)	0.88809 (8)	0.74956 (3)	0.02905 (19)	
F6	−0.05042 (10)	0.98987 (7)	0.68110 (3)	0.02762 (19)	
P2	0.70821 (4)	0.49928 (3)	0.947765 (13)	0.02189 (8)	
F7A	0.5758 (12)	0.4191 (10)	0.9282 (3)	0.078 (2)	0.64 (3)
F8A	0.6652 (12)	0.5050 (9)	0.9993 (4)	0.0473 (18)	0.64 (3)
F9A	0.8492 (8)	0.5779 (7)	0.9694 (3)	0.0372 (10)	0.64 (3)
F10A	0.7584 (13)	0.4898 (3)	0.8965 (2)	0.0429 (14)	0.64 (3)
F7B	0.5535 (12)	0.4370 (13)	0.9296 (4)	0.052 (2)	0.36 (3)
F8B	0.689 (2)	0.5172 (17)	1.0023 (7)	0.050 (3)	0.36 (3)
F9B	0.8528 (13)	0.5644 (17)	0.9618 (8)	0.053 (3)	0.36 (3)
F10B	0.7239 (19)	0.4851 (9)	0.8938 (4)	0.056 (3)	0.36 (3)
F11	0.62602 (11)	0.62115 (9)	0.93069 (4)	0.0452 (3)	
F12	0.79372 (13)	0.37811 (8)	0.96468 (4)	0.0462 (3)	

### Atomic displacement parameters (Å^2^)

**Table:**

	*U*^11^	*U*^22^	*U*^33^	*U*^12^	*U*^13^	*U*^23^
N1	0.0263 (6)	0.0145 (5)	0.0190 (5)	0.0008 (4)	0.0065 (4)	−0.0006 (4)
N2	0.0266 (6)	0.0214 (5)	0.0256 (6)	0.0007 (5)	0.0077 (5)	0.0003 (5)
N3	0.0255 (6)	0.0136 (5)	0.0181 (5)	−0.0010 (4)	0.0040 (4)	0.0006 (4)
N4	0.0256 (6)	0.0331 (7)	0.0261 (6)	−0.0044 (5)	0.0013 (5)	0.0069 (5)
C1	0.0220 (6)	0.0206 (6)	0.0192 (6)	−0.0008 (5)	0.0080 (5)	−0.0014 (5)
C2	0.0231 (6)	0.0169 (5)	0.0221 (6)	0.0001 (5)	0.0075 (5)	0.0001 (5)
C3	0.0312 (7)	0.0176 (6)	0.0268 (7)	0.0000 (5)	0.0133 (6)	−0.0024 (5)
C4	0.0362 (8)	0.0212 (6)	0.0252 (7)	−0.0007 (5)	0.0159 (6)	−0.0009 (5)
C5	0.0352 (8)	0.0182 (6)	0.0269 (7)	−0.0015 (5)	0.0137 (6)	0.0022 (5)
C6	0.0235 (6)	0.0171 (6)	0.0238 (7)	−0.0005 (5)	0.0091 (5)	−0.0021 (5)
C7	0.0249 (7)	0.0180 (6)	0.0278 (7)	−0.0005 (5)	0.0097 (6)	−0.0027 (5)
C8	0.0287 (7)	0.0177 (6)	0.0196 (6)	0.0003 (5)	0.0060 (5)	0.0002 (5)
C9	0.0381 (8)	0.0210 (6)	0.0180 (6)	0.0027 (6)	0.0055 (6)	0.0025 (5)
C10	0.0322 (8)	0.0238 (7)	0.0238 (7)	0.0037 (6)	0.0025 (6)	0.0020 (5)
C11	0.0287 (8)	0.0318 (8)	0.0400 (9)	−0.0006 (6)	0.0160 (7)	0.0007 (7)
C12	0.0237 (7)	0.0362 (8)	0.0298 (8)	0.0016 (6)	0.0078 (6)	−0.0029 (6)
C13	0.0449 (10)	0.0491 (11)	0.0542 (12)	0.0066 (9)	0.0292 (9)	0.0007 (9)
C14	0.0530 (12)	0.0621 (13)	0.0593 (14)	0.0055 (10)	0.0320 (11)	−0.0127 (11)
C15	0.0240 (6)	0.0191 (6)	0.0244 (7)	0.0023 (5)	0.0065 (5)	0.0038 (5)
C16	0.0265 (7)	0.0204 (6)	0.0192 (6)	0.0005 (5)	0.0038 (5)	0.0005 (5)
C17	0.0444 (9)	0.0164 (6)	0.0182 (6)	−0.0023 (6)	0.0022 (6)	−0.0024 (5)
C18	0.0410 (9)	0.0254 (7)	0.0254 (7)	−0.0105 (6)	−0.0039 (6)	0.0028 (6)
C19A	0.0258 (13)	0.0306 (18)	0.0399 (16)	−0.0018 (16)	0.0136 (11)	0.0025 (16)
C20A	0.0234 (12)	0.0317 (13)	0.0454 (19)	−0.0030 (9)	0.0088 (12)	−0.0032 (12)
C21A	0.0245 (11)	0.0269 (12)	0.0288 (11)	−0.0001 (9)	0.0034 (9)	−0.0041 (9)
C22A	0.0267 (13)	0.0304 (13)	0.0396 (16)	0.0037 (10)	0.0005 (13)	−0.0037 (13)
C19B	0.027 (3)	0.025 (3)	0.034 (3)	−0.002 (3)	0.003 (2)	0.001 (3)
C20B	0.019 (2)	0.041 (3)	0.022 (3)	−0.007 (2)	0.002 (2)	−0.002 (2)
C21B	0.023 (2)	0.028 (3)	0.043 (3)	0.004 (2)	0.014 (2)	0.010 (2)
C22B	0.031 (4)	0.038 (4)	0.072 (6)	0.004 (3)	0.000 (4)	0.015 (4)
C23	0.0534 (10)	0.0283 (7)	0.0287 (8)	0.0001 (7)	0.0223 (7)	0.0005 (6)
P1	0.02485 (18)	0.01586 (15)	0.01977 (17)	−0.00042 (12)	0.00237 (13)	−0.00059 (12)
F1	0.0344 (5)	0.0262 (4)	0.0401 (5)	−0.0078 (4)	0.0098 (4)	0.0037 (4)
F2	0.0330 (5)	0.0254 (4)	0.0368 (5)	0.0078 (4)	0.0042 (4)	−0.0055 (4)
F3	0.0323 (4)	0.0242 (4)	0.0245 (4)	0.0060 (3)	0.0028 (3)	−0.0049 (3)
F4	0.0343 (5)	0.0232 (4)	0.0236 (4)	−0.0004 (3)	−0.0055 (4)	−0.0011 (3)
F5	0.0328 (5)	0.0250 (4)	0.0279 (4)	−0.0080 (3)	0.0032 (4)	0.0026 (3)
F6	0.0398 (5)	0.0230 (4)	0.0182 (4)	0.0021 (3)	0.0021 (4)	−0.0010 (3)
P2	0.02675 (18)	0.01888 (16)	0.01996 (17)	−0.00170 (13)	0.00483 (14)	−0.00076 (13)
F7A	0.074 (4)	0.063 (3)	0.079 (3)	−0.047 (3)	−0.024 (2)	0.005 (2)
F8A	0.057 (3)	0.057 (2)	0.037 (4)	0.0006 (17)	0.031 (3)	0.003 (2)
F9A	0.039 (2)	0.0269 (14)	0.0385 (18)	−0.0080 (11)	−0.0080 (11)	−0.0024 (12)
F10A	0.084 (4)	0.0229 (17)	0.0288 (16)	0.0030 (13)	0.0279 (19)	0.0019 (9)
F7B	0.027 (3)	0.067 (4)	0.058 (4)	−0.022 (3)	0.001 (2)	0.029 (3)
F8B	0.068 (7)	0.060 (5)	0.022 (3)	0.024 (5)	0.008 (3)	−0.013 (3)
F9B	0.015 (3)	0.049 (6)	0.088 (8)	−0.007 (3)	−0.005 (4)	0.023 (5)
F10B	0.057 (5)	0.095 (7)	0.021 (2)	0.026 (3)	0.015 (2)	0.011 (3)
F11	0.0385 (6)	0.0373 (5)	0.0541 (7)	0.0151 (4)	−0.0025 (5)	0.0061 (5)
F12	0.0840 (8)	0.0214 (4)	0.0350 (5)	0.0163 (5)	0.0168 (5)	0.0048 (4)

### Geometric parameters (Å, °)

**Table:**

N1—C8	1.3285 (17)	C17—C18	1.349 (2)
N1—C9	1.3828 (18)	C17—H17A	0.9300
N1—C7	1.4753 (17)	C18—H18A	0.9300
N2—C8	1.3272 (18)	C19A—C20A	1.522 (5)
N2—C10	1.3825 (18)	C19A—H19A	0.9700
N2—C11	1.4751 (19)	C19A—H19B	0.9700
N3—C16	1.3238 (17)	C20A—C21A	1.528 (3)
N3—C17	1.3837 (17)	C20A—H20A	0.9700
N3—C15	1.4764 (18)	C20A—H20B	0.9700
N4—C16	1.3238 (18)	C21A—C22A	1.504 (4)
N4—C18	1.378 (2)	C21A—H21A	0.9700
N4—C19B	1.410 (10)	C21A—H21B	0.9700
N4—C19A	1.516 (4)	C22A—H22A	0.9600
C1—C6	1.3938 (18)	C22A—H22B	0.9600
C1—C2	1.3959 (17)	C22A—H22C	0.9600
C1—H1A	0.9300	C19B—C20B	1.521 (9)
C2—C3	1.3954 (18)	C19B—H19C	0.9700
C2—C15	1.5141 (18)	C19B—H19D	0.9700
C3—C4	1.3921 (19)	C20B—C21B	1.532 (8)
C3—H3A	0.9300	C20B—H20C	0.9700
C4—C5	1.3927 (19)	C20B—H20D	0.9700
C4—C23	1.508 (2)	C21B—C22B	1.485 (9)
C5—C6	1.3972 (19)	C21B—H21C	0.9700
C5—H5A	0.9300	C21B—H21D	0.9700
C6—C7	1.5170 (18)	C22B—H22D	0.9600
C7—H7A	0.9700	C22B—H22E	0.9600
C7—H7B	0.9700	C22B—H22F	0.9600
C8—H8A	0.9300	C23—H23A	0.9600
C9—C10	1.351 (2)	C23—H23B	0.9600
C9—H9A	0.9300	C23—H23C	0.9600
C10—H10A	0.9300	P1—F6	1.5941 (9)
C11—C12	1.515 (2)	P1—F1	1.5960 (9)
C11—H11A	0.9700	P1—F2	1.5976 (9)
C11—H11B	0.9700	P1—F4	1.6120 (9)
C12—C13	1.525 (2)	P1—F5	1.6145 (9)
C12—H12A	0.9700	P1—F3	1.6154 (9)
C12—H12B	0.9700	P2—F9B	1.544 (13)
C13—C14	1.512 (3)	P2—F10B	1.556 (11)
C13—H13A	0.9700	P2—F7A	1.557 (6)
C13—H13B	0.9700	P2—F8A	1.583 (9)
C14—H14A	0.9600	P2—F8B	1.584 (16)
C14—H14B	0.9600	P2—F11	1.5959 (10)
C14—H14C	0.9600	P2—F12	1.6024 (10)
C15—H15A	0.9700	P2—F10A	1.610 (6)
C15—H15B	0.9700	P2—F9A	1.620 (7)
C16—H16A	0.9300	P2—F7B	1.622 (10)
			
C8—N1—C9	108.18 (12)	H20A—C20A—H20B	107.5
C8—N1—C7	124.42 (12)	C22A—C21A—C20A	112.2 (2)
C9—N1—C7	127.40 (12)	C22A—C21A—H21A	109.2
C8—N2—C10	108.38 (12)	C20A—C21A—H21A	109.2
C8—N2—C11	124.06 (13)	C22A—C21A—H21B	109.2
C10—N2—C11	127.46 (13)	C20A—C21A—H21B	109.2
C16—N3—C17	108.54 (12)	H21A—C21A—H21B	107.9
C16—N3—C15	124.05 (11)	N4—C19B—C20B	103.1 (5)
C17—N3—C15	127.23 (12)	N4—C19B—H19C	111.2
C16—N4—C18	108.47 (13)	C20B—C19B—H19C	111.2
C16—N4—C19B	133.8 (4)	N4—C19B—H19D	111.2
C18—N4—C19B	116.9 (4)	C20B—C19B—H19D	111.2
C16—N4—C19A	121.1 (2)	H19C—C19B—H19D	109.1
C18—N4—C19A	129.8 (2)	C19B—C20B—C21B	113.3 (6)
C19B—N4—C19A	19.6 (3)	C19B—C20B—H20C	108.9
C6—C1—C2	120.31 (12)	C21B—C20B—H20C	108.9
C6—C1—H1A	119.8	C19B—C20B—H20D	108.9
C2—C1—H1A	119.8	C21B—C20B—H20D	108.9
C3—C2—C1	119.25 (12)	H20C—C20B—H20D	107.7
C3—C2—C15	120.34 (12)	C22B—C21B—C20B	110.8 (7)
C1—C2—C15	120.41 (12)	C22B—C21B—H21C	109.5
C4—C3—C2	121.32 (12)	C20B—C21B—H21C	109.5
C4—C3—H3A	119.3	C22B—C21B—H21D	109.5
C2—C3—H3A	119.3	C20B—C21B—H21D	109.5
C3—C4—C5	118.60 (13)	H21C—C21B—H21D	108.1
C3—C4—C23	120.58 (13)	C21B—C22B—H22D	109.5
C5—C4—C23	120.77 (13)	C21B—C22B—H22E	109.5
C4—C5—C6	121.11 (13)	H22D—C22B—H22E	109.5
C4—C5—H5A	119.4	C21B—C22B—H22F	109.5
C6—C5—H5A	119.4	H22D—C22B—H22F	109.5
C1—C6—C5	119.40 (12)	H22E—C22B—H22F	109.5
C1—C6—C7	120.42 (12)	C4—C23—H23A	109.5
C5—C6—C7	120.14 (12)	C4—C23—H23B	109.5
N1—C7—C6	111.93 (11)	H23A—C23—H23B	109.5
N1—C7—H7A	109.2	C4—C23—H23C	109.5
C6—C7—H7A	109.2	H23A—C23—H23C	109.5
N1—C7—H7B	109.2	H23B—C23—H23C	109.5
C6—C7—H7B	109.2	F6—P1—F1	90.75 (5)
H7A—C7—H7B	107.9	F6—P1—F2	91.01 (5)
N2—C8—N1	109.20 (12)	F1—P1—F2	91.48 (5)
N2—C8—H8A	125.4	F6—P1—F4	179.23 (6)
N1—C8—H8A	125.4	F1—P1—F4	89.69 (5)
C10—C9—N1	107.24 (12)	F2—P1—F4	89.61 (5)
C10—C9—H9A	126.4	F6—P1—F5	90.17 (5)
N1—C9—H9A	126.4	F1—P1—F5	178.60 (5)
C9—C10—N2	107.00 (13)	F2—P1—F5	89.56 (5)
C9—C10—H10A	126.5	F4—P1—F5	89.38 (5)
N2—C10—H10A	126.5	F6—P1—F3	90.33 (5)
N2—C11—C12	112.49 (13)	F1—P1—F3	89.61 (5)
N2—C11—H11A	109.1	F2—P1—F3	178.26 (5)
C12—C11—H11A	109.1	F4—P1—F3	89.04 (5)
N2—C11—H11B	109.1	F5—P1—F3	89.34 (5)
C12—C11—H11B	109.1	F9B—P2—F10B	91.4 (7)
H11A—C11—H11B	107.8	F9B—P2—F7A	170.0 (7)
C11—C12—C13	111.65 (14)	F10B—P2—F7A	81.0 (6)
C11—C12—H12A	109.3	F9B—P2—F8A	98.5 (9)
C13—C12—H12A	109.3	F10B—P2—F8A	170.0 (7)
C11—C12—H12B	109.3	F7A—P2—F8A	89.3 (5)
C13—C12—H12B	109.3	F9B—P2—F8B	88.8 (9)
H12A—C12—H12B	108.0	F10B—P2—F8B	178.3 (8)
C14—C13—C12	112.36 (16)	F7A—P2—F8B	99.0 (8)
C14—C13—H13A	109.1	F8A—P2—F8B	9.7 (11)
C12—C13—H13A	109.1	F9B—P2—F11	92.0 (7)
C14—C13—H13B	109.1	F10B—P2—F11	86.7 (4)
C12—C13—H13B	109.1	F7A—P2—F11	94.1 (5)
H13A—C13—H13B	107.9	F8A—P2—F11	91.6 (4)
C13—C14—H14A	109.5	F8B—P2—F11	91.6 (7)
C13—C14—H14B	109.5	F9B—P2—F12	86.9 (7)
H14A—C14—H14B	109.5	F10B—P2—F12	92.8 (4)
C13—C14—H14C	109.5	F7A—P2—F12	86.9 (5)
H14A—C14—H14C	109.5	F8A—P2—F12	89.1 (4)
H14B—C14—H14C	109.5	F8B—P2—F12	88.9 (7)
N3—C15—C2	110.71 (11)	F11—P2—F12	178.80 (7)
N3—C15—H15A	109.5	F9B—P2—F10A	80.3 (7)
C2—C15—H15A	109.5	F10B—P2—F10A	11.7 (7)
N3—C15—H15B	109.5	F7A—P2—F10A	91.7 (4)
C2—C15—H15B	109.5	F8A—P2—F10A	177.3 (5)
H15A—C15—H15B	108.1	F8B—P2—F10A	168.9 (8)
N3—C16—N4	109.00 (12)	F11—P2—F10A	90.8 (2)
N3—C16—H16A	125.5	F12—P2—F10A	88.5 (2)
N4—C16—H16A	125.5	F9B—P2—F9A	9.4 (10)
C18—C17—N3	106.69 (13)	F10B—P2—F9A	99.8 (7)
C18—C17—H17A	126.7	F7A—P2—F9A	177.6 (5)
N3—C17—H17A	126.7	F8A—P2—F9A	90.0 (5)
C17—C18—N4	107.30 (13)	F8B—P2—F9A	80.3 (9)
C17—C18—H18A	126.4	F11—P2—F9A	88.3 (3)
N4—C18—H18A	126.4	F12—P2—F9A	90.8 (3)
N4—C19A—C20A	111.9 (3)	F10A—P2—F9A	88.9 (4)
N4—C19A—H19A	109.2	F9B—P2—F7B	175.9 (8)
C20A—C19A—H19A	109.2	F10B—P2—F7B	85.8 (7)
N4—C19A—H19B	109.2	F7A—P2—F7B	10.6 (9)
C20A—C19A—H19B	109.2	F8A—P2—F7B	84.3 (6)
H19A—C19A—H19B	107.9	F8B—P2—F7B	93.9 (9)
C19A—C20A—C21A	114.8 (3)	F11—P2—F7B	84.9 (5)
C19A—C20A—H20A	108.6	F12—P2—F7B	96.2 (5)
C21A—C20A—H20A	108.6	F10A—P2—F7B	97.1 (5)
C19A—C20A—H20B	108.6	F9A—P2—F7B	170.9 (6)
C21A—C20A—H20B	108.6		
			
C6—C1—C2—C3	−1.1 (2)	N2—C11—C12—C13	170.61 (14)
C6—C1—C2—C15	178.02 (13)	C11—C12—C13—C14	−176.02 (17)
C1—C2—C3—C4	0.4 (2)	C16—N3—C15—C2	90.70 (15)
C15—C2—C3—C4	−178.69 (14)	C17—N3—C15—C2	−83.84 (16)
C2—C3—C4—C5	0.3 (2)	C3—C2—C15—N3	92.95 (15)
C2—C3—C4—C23	−177.08 (15)	C1—C2—C15—N3	−86.17 (15)
C3—C4—C5—C6	−0.3 (2)	C17—N3—C16—N4	0.59 (15)
C23—C4—C5—C6	177.02 (15)	C15—N3—C16—N4	−174.83 (11)
C2—C1—C6—C5	1.1 (2)	C18—N4—C16—N3	−0.45 (16)
C2—C1—C6—C7	178.94 (12)	C19B—N4—C16—N3	−169.1 (5)
C4—C5—C6—C1	−0.3 (2)	C19A—N4—C16—N3	171.83 (19)
C4—C5—C6—C7	−178.22 (14)	C16—N3—C17—C18	−0.49 (15)
C8—N1—C7—C6	−101.42 (15)	C15—N3—C17—C18	174.74 (12)
C9—N1—C7—C6	79.54 (16)	N3—C17—C18—N4	0.22 (16)
C1—C6—C7—N1	86.62 (16)	C16—N4—C18—C17	0.13 (17)
C5—C6—C7—N1	−95.51 (15)	C19B—N4—C18—C17	171.0 (4)
C10—N2—C8—N1	1.08 (16)	C19A—N4—C18—C17	−171.3 (2)
C11—N2—C8—N1	177.63 (12)	C16—N4—C19A—C20A	−105.3 (3)
C9—N1—C8—N2	−0.99 (15)	C18—N4—C19A—C20A	65.2 (3)
C7—N1—C8—N2	179.81 (11)	C19B—N4—C19A—C20A	119.3 (17)
C8—N1—C9—C10	0.52 (16)	N4—C19A—C20A—C21A	61.1 (4)
C7—N1—C9—C10	179.69 (12)	C19A—C20A—C21A—C22A	−175.4 (3)
N1—C9—C10—N2	0.13 (16)	C16—N4—C19B—C20B	−72.2 (7)
C8—N2—C10—C9	−0.73 (16)	C18—N4—C19B—C20B	119.9 (5)
C11—N2—C10—C9	−177.13 (14)	C19A—N4—C19B—C20B	−15.9 (11)
C8—N2—C11—C12	−105.44 (16)	N4—C19B—C20B—C21B	−60.3 (7)
C10—N2—C11—C12	70.44 (19)	C19B—C20B—C21B—C22B	169.3 (6)

### Hydrogen-bond geometry (Å, °)

**Table:**

Cg1 is the centroid of the C1–C6 phenyl ring.

**Table:**

*D*—H···*A*	*D*—H	H···*A*	*D*···*A*	*D*—H···*A*
C8—H8A···F4	0.93	2.42	3.0154 (16)	121
C8—H8A···F5	0.93	2.39	3.2700 (16)	157
C16—H16A···F3	0.93	2.31	3.1941 (16)	160
C16—H16A···F4	0.93	2.45	3.1677 (16)	134
C21A—H21B···F4	0.97	2.49	3.196 (2)	130
C3—H3A···F12^i^	0.93	2.44	3.3147 (16)	157
C5—H5A···F9A^ii^	0.93	2.55	3.420 (8)	156
C7—H7A···F10A^ii^	0.97	2.51	3.427 (6)	158
C9—H9A···F12^iii^	0.93	2.48	3.2805 (17)	144
C12—H12A···F3^iv^	0.97	2.53	3.3358 (18)	140
C18—H18A···F8A^v^	0.93	2.38	3.148 (11)	140
C22A—H22C···Cg1^vi^	0.96	2.76	3.535 (3)	138
C23—H23B···Cg1^vii^	0.96	2.59	3.487 (2)	155
C22B—H22E···Cg1^vi^	0.96	2.86	3.637 (8)	139

Symmetry codes: (i) *x*−1, *y*+1, *z*; (ii) *x*−1, *y*, *z*; (iii) −*x*+1, −*y*+1, −*z*+2; (iv) −*x*, *y*−1/2, −*z*+3/2; (v) −*x*+1, −*y*+2, −*z*+2; (vi) *x*+1, *y*, *z*; (vii) −*x*, −*y*+2, −*z*+2.
